# Recent Advanced Metabolic and Genetic Engineering of Phenylpropanoid Biosynthetic Pathways

**DOI:** 10.3390/ijms22179544

**Published:** 2021-09-03

**Authors:** Muhammad Anwar, Liu Chen, Yibo Xiao, Jinsong Wu, Lihui Zeng, Hui Li, Qingyu Wu, Zhangli Hu

**Affiliations:** 1 Guangdong Technology Research Center for Marine Algal Bioengineering, Guangdong Key Laboratory of Plant Epigenetics, College of Life Sciences and Oceanography, Shenzhen University, Shenzhen 518060, China; postdoc@szu.edu.cn (M.A.); liuchenneo@163.com (L.C.); yiboxiao@szu.edu.cn (Y.X.); lihui80@szu.edu.cn (H.L.); qingyu@tsinghua.edu.cn (Q.W.); 2 Key Laboratory of Optoelectronic Devices and Systems of Ministry of Education and Guangdong Province, College of Optoelectronic Engineering, Shenzhen University, Shenzhen 518060, China; 3 Shenzhen Key Laboratory of Marine Bioresource & Eco-Environmental Science, Longhua Innovation Institute for Biotechnology, Shenzhen University, Shenzhen 518060, China; 1800252001@email.szu.edu.cn; 4 College of Horticulture, Fujian Agriculture and Forestry University, Fuzhou 350002, China; lhzeng@fafu.edu.cn

**Keywords:** tanshinones, phenolic acid, plant defense, pdLNLD/ELxiG/S motif, flavonoids, repressor MYB, bioactive compounds

## Abstract

The MYB transcription factors (TFs) are evolving as critical role in the regulation of the phenylpropanoid and tanshinones biosynthetic pathway. MYB TFs relate to a very important gene family, which are involved in the regulation of primary and secondary metabolisms, terpenoids, bioactive compounds, plant defense against various stresses and cell morphology. R2R3 MYB TFs contained a conserved N-terminal domain, but the domain at C-terminal sorts them different regarding their structures and functions. MYB TFs suppressors generally possess particular repressive motifs, such as pdLNLD/ELxiG/S and TLLLFR, which contribute to their suppression role through a diversity of complex regulatory mechanisms. A novel flower specific “NF/YWSV/MEDF/LW” conserved motif has a great potential to understand the mechanisms of flower development. In the current review, we summarize recent advanced progress of MYB TFs on transcription regulation, posttranscriptional, microRNA, conserved motif and propose directions to future prospective research. We further suggest there should be more focus on the investigation for the role of MYB TFs in microalgae, which has great potential for heterologous protein expression system for future perspectives.

## 1. Introduction

The compounds derived from phenylpropanoid denote a different class of secondary metabolites, which start from key enzyme phenylalanine. Phenylpropanoid derived metabolites play an important function in plant resistance mechanisms against biotic and abiotic stress, regulate plant growth and development [1,2] and male fertility [3]. Several of these phenylpropanoid derived compounds are considered to be valuable to human welfare and health. MYB protein associated with a big class of transcription factors, which are responsible for the regulation of the biosynthetic pathway of phenylpropanoid resulting compounds [4]. In plants, phenylpropanoid derived secondary metabolites mainly consist of flavonoid, monolignol, stilbenes, terpenoids and different phenolic acid. Many of these compounds play a key role in identified plants, including as UV light protectants, phytoalexins, carotenoids, strengthen the cell wall and signaling molecules [5].

The pigments that provide the different colors to vegetables, fruits, ornamental foliage, leaves, ornamental flowers and seeds are called flavonoids, which provide health benefits to the human and animals [6]. Flavonoids are the secondary metabolites that are broadly distributed in the plant kingdom, which play key roles in plant defense and development. These secondary metabolites can be divided into different groups based on differences in their structure, such as anthocyanin, proanthocyanin, chalcones, flavones, flavonols, flavandiols, isoflavonoids and phlobaphenes [7]. Flavonoids are the most common occurring pigment in plants. Anthocyanins are commonly known as flavonoid compounds providing blues, pink hues, orange, yellow and red colors to flowers, fruits and vegetables. Anthocyanins play very significant physiological and ecological roles in plants. Anthocyanin is most noticeable in young leaves where they defend developing tissues from light stress. Anthocyanins play a key role in seed dispersal and pollination by attracting the pollinator agent in mature fruits and flowers. Proanthocyanin (also known as tannins) provides as significantly roles, such as strengthening the seed coat and stress tolerance in plants [8]. Furthermore, these compounds are concerned in the regulation signaling, when legume are in nodulation process, transportation of auxin and male fertility determination. Moreover, these compounds are involved in plant defense in opposition to stress (biotic and abiotic). These compounds have very imperative values as nutritional and pharmaceutical compounds [9].

**Figure 1:**
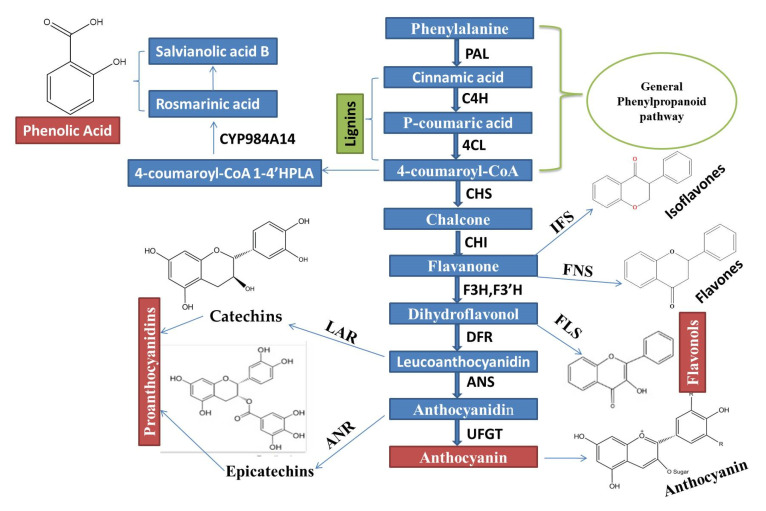
An Integrated regulatory mechanisms network of the Phenylpropanoid biosynthetic pathway, which is controlled through MYB TFs and MBW complex activation, is constructed based on the recent remarkable research advancement.

Phenylalanine ammonia-lyase (*PAL*), cinnamate 4-hydroxylase (*C4H*), and p-coumaroyl coenzyme A ligase (*4CL*) are very important key enzymes, which jointly catalyzed stages (First three stages) involved in biosynthesis of compounds, which are derived from phenylpropanoid, as shown in (Figure 1).

Various transcription factors (TFs), including R2R3 MYB, WD40 repeat (WDR) proteins and basic helix-loop-helix (*bHLH*) and control the biosynthesis of flavonoid compounds [10]. A complex of MYB-bHLH-WDR (MBW) shows action, in order to trigger the structural genes responsible for the process of flavonoid biosynthesis. In several plants, including *Helianthus annuus* L., *Arabodopsis thialana*, *Mimulus guttatus*, *Camellia sinensis*, *Narcissus tazetta.* L. *Narcissus tazetta*, *Zea mays*, *Glycine max*, *Medicago truncatula*, *Fragaria × ananassa*, populous, *Petunia x hybrida*, *Malus domestica*, and *Vitis vinifera* L., these transcription factors have been functionally characterized well [11,12,13,14,15,16,17,18,19]. This analysis encapsulates the recent understanding of MYB proteins and their function in controlling phenylpropanoid metabolisms in plants, as well as further studies to understand the complexity of their network of regulatory mechanisms.

## 2. Mechanisms of MYB Gene Family as a Transcription Factor

Cellular processes are regulated by transcription factors (TFs), which can modify complex or intricate traits in plants and could play a prominent part in next-generation biotechnology. There are limitations in genomic diversity in traditional breeding. However, transgenic methodologies surpass genetic obstacles by improving the regulatory pathways of one crop by integrating TFs of other crop or plant species [20]. Genes that encode TFs containing DNA binding motifs, e.g., MYB, ERF/AP2, Zinc fingers and bZIP are signal-induced. These TFs further regulate many functional genes during different conditions of stress or morphogenesis. Therefore, identifying novel TF genes responsible for regulating particular gene expression will improve our understating of signaling pathways related to the development and growth of innovative transgenic crops. MYB is a functionally diverse and large protein family present in all eukaryotic organisms [21]. Many MYB acts as TFs having a different number of MYB domains that are able to bind DNA. They interact with other TFs and are also involved in ABA response, which represents their wide distribution among plants. Detailed functional characterization of these proteins in *Arabidopsis thaliana* depicts their variety of roles in plant-specific mechanisms. The cell cycle of eukaryotes is controlled by ‘classical’ MYB factors that are linked with c-Myb. First, MYB gene identification was form avian virus myeloblastosis, which was ‘oncogene’ v-MYB [22].

## 3. Evolution of MYB Transcription Factors

The protein of MYB family contains DNA binding domain. There are two particular conserved regions present in MYB protein, C-terminal of R2R3 MYB protein, which show structural and functional diversity in their amino acid sequence, which are responsible for various regulation activities in plants. While, N-terminal show binding domain of MYB DNA are conserved. Generally, the domain of MYB protein comprises sequences with four imperfect amino acid repeats of approximately 52 amino acids, each establishing three α–helices. Ogata, et al. [23] described that Helix-turn-helix structure, which are built through each repeat of second and third helices with regularly spaced three tryptophan residues, resulting in hydrophobic central in HTH structure (3D). Interestingly, first tryptophan in R3 domain is replaced with isoleucine or phenylalanine in plants. MYB family could be separated into four group based on MYB domain number [2,24]. In plants (monocots and dicots), plentiful kind of R2R3-MYB TFs are specific [25]. The plant taxon represents the highest diversity, with the presence of all four classes of MYB proteins. The group of 4R-MYB indicates the smallest class and its members have four R1/R2-type repeats. Several plant genomes contain single 4R-MYB encoded protein. However, the second class retains 3R-MYB protein of R1R2R3 type, which is composed of higher plant genomes, is particularly encoded by five genes. R2R3-MYB domain is more conserved as compared to its other region, which shows more divergence. The division of R2R3-MYB proteins into subgroups is based on amino acid motifs, which are present at C terminal [2].

MYB domain sequence-based evolutionary studies from various organisms represent that plant ancestor initially had three repeats and out of which the first repeat was lost during the course of time. Lipsick [21] has described an evolutionary model of MYB proteins. This model reveals that R1R2R3-MYBs resulted due to consecutive intragenic duplications and triplications among the primeval eukaryotes, and they produced two repeat and three repeat (R1R2R3-MYB, R2R3-MYB) proteins in animals and plants, respectively. During plant evolution through selective subgroup expansion and amplification, numerous subgroups genes harboring R2R3MYB proteins were made due to the loss of R1 [26]. The consecutive gain of repeat units generated MYB genes. The detailed study of MYB genes regarding their classification, structure, characteristics, mechanism of combinational control, multi-functionality, functional redundancy and gain model for evolution have been reviewed comprehensively by Du, et al. [27] and Dubos, Stracke, Grotewold, Weisshaar, Martin and Lepiniec [24]. It is very interesting that the heterogeneous class consists of proteins with partial or single MYB repeat, jointly known as “MYB-related”, which is further divided into many subclasses [28]. The loss of sequence regarding R1 repeat and successive extension of gene family resulted in R2R3-MYB class after evolution from R1R2-MYB gene predecessor [28].

Moreover, it has also been proposed that ancient intragenic duplication by gaining the sequence encoding R1 repeat from R2R3-MYB genes resulted in the evolution of 3R-MYB [29]. Arabidopsis *AtMYB48* and *AtMYB59* and their rice homologs (*OsMYBAS2* and *OsMYBAS1*), the two R2R3-MYB genes experience alternative splicing in the same way and result in three diverse merged transcripts in rice, and four in Arabidopsis. Therefore, a deep-rooted understanding of another splicing of MYB protein will further enlighten us regarding gene evolution in dicots and monocots, as well as development-related regulation by transcription factor genes [29].

## 4. Recent Transcriptomic and Genome-Wide Analysis and Expression of MYB Transcription Factors

In sugarcane, 202 MYB TFs are explored, some of them are expressed mainly in stem and are actively responded to drought stress resistance and mosaic diseases [30]. In Arabidopsis 198 MYBs have been identified; among them, 126 are encoded for R2R3-MYB proteins [31]. Recently, 223 MYB (112 R2R3-MYB, 2 R1R2R3-MYB and 119 R1-MYB) transcription factors were recognized in the potato genome [32]. Recently, there are 69 *GbMYB* transcription factors are identified in *Ginkgo biloba*, out of which 19 R2R3 MYB are responsive to hormonal and abiotic stresses [33]. In maize, a genome-wide survey indicated that they consist of 157 R2R3-MYB proteins [34]. R2R3-MYB (185) transcription factors are reported in the genome of Mangrove, 34 MYB gene are mainly expressed in different tissues (root, leaves), which are related to various stresses (salinity and drought) [35]. *Hippophae rhamnoides* is the rich source of secondary metabolites, which has economic importance regarding medicinal and nutritional values, 161 R2R3–MYB TFs were obtained through its genome-wide analysis [36]. In a recent study, 111 StR2R3-MYB transcription factors are reported in potato [37]. In the genome of flax, 167 R2R3-MYB, 7 R3-MYB, and 1R4-MYB transcription factors have been identified [38]. However, in soybean, 252 total MYBs were identified and account for about 4% of all transcription factors. They consist of two (4R-MYB) genes, six (3R-MYB) proteins and 244 encodings for R2R3-MYB proteins [39]. Genome-wide analysis of apples revealed that they contained 229 MYB transcription factors. Another recent study has explored 251 and 305 MYB TFs from *Musa balbisiana* and *Musa acuminata*, respectively by [40].

## 5. Biological Functions Regulated through MYB Transcription Factors

MYB TFs control many plant-specific processes. By using molecular and genetic analysis the function of MYB proteins have been greatly described among various plant species, like petunia (*Petunia hybrida*), apple (*Malus domestica*), poplar (*Populus tremuloides*), snapdragon (*Antirrhinum majus*), grapevine (*Vitis vinifera* L.), maize (*Zea mays*) and *Arabidopsis thaliana* [41]. R2R3-MYB TFs have been widely investigated during last decade and their involvement in several processes have been revealed, such as abiotic and biotic stress [42,43], cold tolerance [41], phenylpropanoid metabolism [44,45], trichomes development [46], flower shape [47], cell shape [48], plant defense mechanisms [49,50,51,52], cell wall development [53] and stomatal closure [54].

## 6. MYB Transcription Factors and Plant Defense Mechanisms

The growth and development of plants are strongly affected by different stresses, including extreme temperature, drought, salinity and cold stress. Several transcriptions factors facilitate stress responses in plants, such as NAC, WRKY, bZIP and MYB. MYB family among them is considered the largest families of transcription factors. MYB protein link to various cis-acting motifs, such as MBSI (T/C)AAC(G/T)G(A/C/T)(A/C/T), MBSII (A/G)(G/T)T(A/T)GGT(A/G), MBSIIG ACC(A/T)ACC(A/C/T), which are associated to resistance to low temperature and cell cycle control, secondary cell wall biosynthesis and flavonoid biosynthesis respectively. MYBCORE, CTGTTG, CAGTTA, which are involved in drought tolerance. MYBs in plants regulate abiotic responses, for example, BplMYB46, improve osmatic and salt tolerance in *Betula platyphylla* by influencing the *SOD* and *POD* genes, to improve both proline levels and reactive oxygen species scavenging, and reduced water loss by regulating stomatal aperture [55]. *GhMYB4*, transcription factors induce resistance against *verticillium dahlia* in cotton. It provide a great potential for the improvement in breeding of cotton plants [56]. High temperature induces the MYB transcriptional factor and positive regulator of thermotolerance [54]. *MdMYB308L* positively regulate anthocyanin accumulation and cold tolerance in apple by interacting with *MdbHLH33* [41]. *StMYB030*, which is the homology of *AtMYB44*, increased the salt stress tolerance in transgenic plants of Arabidopsis upon its overexpression [32]. *VdMYB1* from Chinese wild grape stimulates defense response against pathogen attack [52]. *GhMYB108-like* plays a key regulating role in response to salt and drought stresses [57,58]. The over-expression of *GmMYB81* in *Arabidopsis thaliana* increases the rates of seed germination under drought and salts stress [59]. *EaMYB18*, was isolated from sugarcane, encoding a single R3 repeat MYB DNA binding domain, showed the highest potential for cold and drought stress tolerance [58]. The overexpression of *OsMYB-R1* in transgenic rice increased tolerance under *Cr(V1)* and drought exposure [60]. *OsMYB30* regulates the expression of *OsPAL6* and *OsPAL*, which play an important role in providing resistance in rice against brown planthopper [61] (see Table 1).

## 7. Regulation Mechanism of Flavonoid Biosynthesis Pathway through MYB Transcription Factors

The flavonoid biosynthetic pathway associated genes are controlled by the collaboration of various families of TFs. The genes responsible for anthocyanin biosynthesis are differentially controlled in monocot and dicots by the MBW complex, which is formed by the physical interaction of R2R3-MYB, bHLH, and WD40 Proteins. This MBW complex stimulates the temporal and spatial expression of structural genes encoding for the biosynthesis of anthocyanin. Anthocyanin biosynthesis controlling in monocot differs from dicots species. MYB and bHLH protein in maize are determined by *Pl*/*C1* and *B*/*R* families, each member of these families has tissues specific pattern. A WD40 transcriptional factor, PAC1 is needed by either B1 or R1 proteins to stimulate the biosynthesis of anthocyanin genes in different tissues (seeds and roots) [66]. In *Arabidopsis thaliana*, *TT2*, *TT8* and *TTG1* activate PA biosynthesis in seeds growth. Whereas *TTG1*, a *WD40* protein, various bHLH and *PAP1*, *PAP2* (MYB) physically interact each other to motivate anthocyanin biosynthesis in vegetative section [67,68].

**Figure 2:**
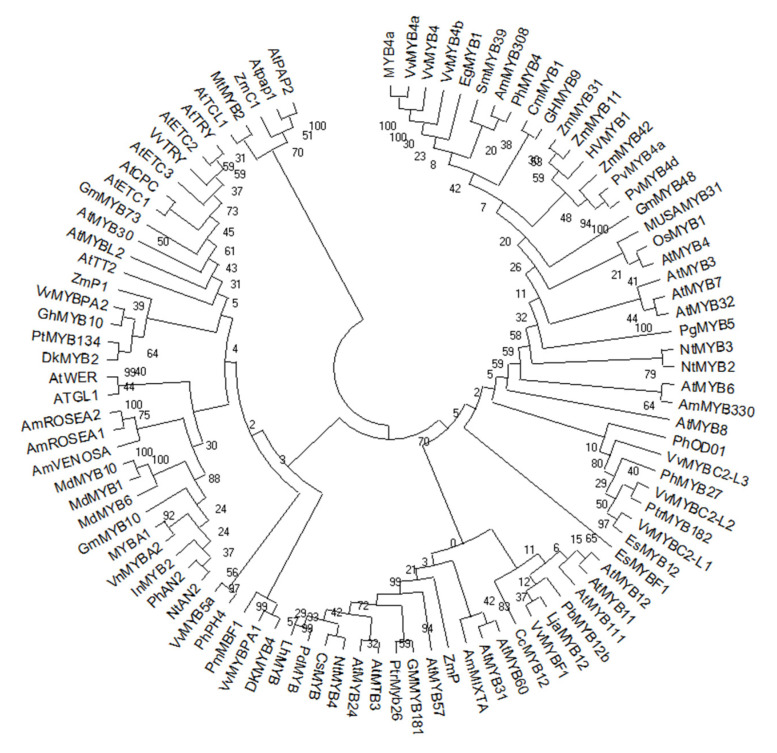
Phylogenetic tree of MYB TFs, which are involved in the regulation of secondary metabolites.

Various R2R3 MYB (TFs) were recognized from many model plants, including *Arabidopsis thaliana* and *Zea mays* are take part in the control of the flavonoid and phenylpropanoid biosynthetic pathway [2,66,69]. Recently plant genome-wide surveys provide the opportunities for the identification and isolation of many MYB TFs responsible for the regulation of flavonoid biosynthesis form different plant species, including strawberry, apple, potato, pear, bayberry, grapevine, pear, poplar, purple kale, soybean and cauliflower (Figure 2) [70]. Most of these MYB genes have been functionally characterized by overexpression in host species.

The anthocyanins exhibited high levels of biological function in plants. It acts as a visual signal to pollinators and provides defense against stresses (biotic and abiotic), including cold tolerance, infection by pathogen, high intensity of light and oxidative damage in plant cells [71,72,73]. Dietary consumption of anthocyanin has been connected with protection against a broad spectrum of human diseases [74]. Therefore, a high level of anthocyanin accumulation and control are needed for economic as well as scientific significance. R2R3-MYB, bHLH and WD-repeat protein act together to form MBW complex. This transcriptional complex is responsible for anthocyanin biosynthesis regulation. In Eudicots, this development starting in stressed leaves and developing flowers by R2R3 MYB proteins activation. Anthocyanins biosynthesis pathway is a branch of flavonoid pathway that has been extensively studied in petunia (*Petunia hybrida*) [17], Lily (Asiatic hybrid lilies) [75], Chinese narcissus [12], monkey flower (*Mimulus*) [76], *Anthurium andraeanum* [77] and *Cymbidium hybrid* [78]. In fruits and vegetables, MYB TFs are also well-described in anthocyanin biosynthesis, such as apple and potato [41,79]. The anthocyanin biosynthetic pathway includes structural as well as regulatory genes. Several recent studies indicated that MBW complex activate the expression of structural genes, which are responsible for the accumulation of anthocyanin pathway. R2R3-MYB proteins in the MBW complex generally take part in the accumulation of anthocyanin [80]. In our previous study, *NtMYB3* and *NtMYB2* are isolated from Chinese narcissus, which are responsible for the regulation of anthocyanin biosynthesis. Heterologous overexpression of *NtMYB2* and *NtMYB3* reduced the anthocyanin contents and down-regulate the expression level of genes, including *UFGT*, *ANS* and *DFR* in the transgenic flowers of tobacco [11,12].

## 8. The Role of Condensed Tannins in Plants and MYB

Condensed tannins (proanthocyanidins) are well-known polyphenols with different ecological functions. It is the polymers of flavan-3-ols and the resultant product of flavonoid pathway [81]. PA is the most extensively spread secondary metabolites and is mainly prominent in forest trees and woody plants [82]. In trees, proanthocyanidins are general constituents of vegetative parts which consist of flowers, leaves, bark, seed and roots [83], and provide protection to plants from various abiotic and biotic stressors. The occurrence of proanthocyanidins (PAs) in herbaceous plants is more limited, while they are found in *lotus corniculatus* and *Onobrychis viciifolia* [84,85]. The accumulations of PAs were observed in seed coat or testa in *Glycine max* and *Arabidopsis thaliana.* In *Brassica napus*, PAs are down-regulated in yellow seed [86]. *PAs* are also found to be accumulate in monocot species such Chinese narcissus [87,88]. PAs have diverse biological functions; they are functionally defined by their capacity to attach and precipitate proteins in solutions, act as antioxidants and as pro-oxidants and provide tolerance to environmental stresses [89,90]. In our previous study, *NtMYB3* and *NtMYB2* are isolated from Chinese narcissus are involved in the regulation of proanthocyanin biosynthesis. The ectopic overexpression of *NtMYB2* reduced the PA in transgenic flowers of tobacco by regulating the main key genes *LAR* and *ANR*. *NtMYB3* positively regulates the transcript level of *ANR* and *LAR* in transgenic tobacco. The PA contents were higher in *NtMYB3* overexpression tobacco flowers as compared to wild [11,12]. In vertebrate herbivores with the naturally acidic stomach, PA attaches nutritional protein and show anti-nutritive effects when found in high concentrations. The forage legumes with balance PAs concentration reduced the risk of rumen foaming and bloating diseases in grazing cattle [91]. FhMYB5 belong to VvMYB5b subclade accumulate proanthocyanidin and anthocyanin in *Freesia hybrida* by up-regulating the *DFR* gene [92]. In ruminants, methane emissions and nematode burden can be minimized by the action of proanthocyanin [93]. Furthermore, the induction of proanthocyanin biosynthesis plays a key role in plant defense [94].

## 9. Physiological and Metabolic Regulation of Phenolic Acid and Terpenoids through MYB Transcription Factors

Phenolic acids are universal secondary metabolites in plants, which play a very important physiological and metabolic role in the entire plant life cycle [95]. Phenolics regulate the various physiological process, which is associated with plant development and growth, cell division, seed germination and photosynthetic pigmentation [96]. Plant shows increased biosynthesis of phenolic and flavonoids under the conditions of abiotic stress, which support the plant to cope with environmental constraints. Phenylpropanoid biosynthetic pathway is stimulated under the condition of abiotic stress, which facilitates the accumulation of different phenolic compounds that have the capacity to scavenge harmful reactive oxygen species. Depending on their carbon skeleton, it can be divided into two main groups, such as the hydroxycinnamic acid group and the hydroxybenzoic acid group. Such compounds are of great medicinal significance; several of them are effective antioxidants, and many others are recognized as anti-inflammatory, anticarcinogenic, antiviral and antibacterial functions [97,98]. MYB TFs are the most significant gene family, which regulate the phenolic biosynthetic pathway as previously identified in various species. *PAL*, *C4H* and *4CL* are very important enzymatic genes, which participate in the polyphenolic biosynthetic pathway (Figure 1). Ding, et al. [99] described that when *SmMYB36* overexpressed in *Salvia miltiorrhiza* (hairy roots), it stimulated the accumulation of tanshinones, but repressed the flavonoid and phenolic acid biosynthesis. A novel gene *SnMYB2* increased the biosynthesis of salvianolic acid in the roots of salvia, which is the potential medicinal herb [100]. *SmMYB1*, which are responsive to methyl jasmonate, enhance the biosynthesis of phenolic acid [101]. *SmMYB2* is another novel gene that regulates the salvianolic acid in *Salvia miltiorrhiza*, and enhanced its biosynthesis [100]. When *AtPAP1* overexpressed in *Brassica napus* increases the antioxidant and sinapic acid content of the leaves. Furthermore, the expression level of genes participated in flavonoid and a phenolic acid biosynthetic pathway were stimulated [102]. *ZmMYB-IF35*, from maize, increase chlorogenic and ferulic acid accumulation [103]. *AtMYB4*, identified from *Arabidopsis thaliana*, belongs to repressor R2R3-MYB subgroup 4, increased the expression level of *C4H* leads to accumulation of sinapate ester in *Atmyb4* mutants [104]. Heterologous expression of ROSEA1(snapdragon) and PAP1(*Arabidopsis thaliana*) increases the level of salvianolic acid and rosmarinic acid [105,106]. The overexpression of *SmMYB39* (subgroup 4) dramatically reduced the total phenolics and contents of rosmarinic acid, salvianolic acid and 4-coumaric acid, in transgenic lines of *Salvia miltiorrhiza by* down-regulating the *C4H* gene. Furthermore, all of these compounds were rescued, when *SmMYB39* was silenced by RNAi [107]. In a recent study, *SmMYB2* improved salvianolic acid biosynthesis in *Salvia miltiorrhiza*, which is a very potential medicinal herb [100].

**Figure 3:**
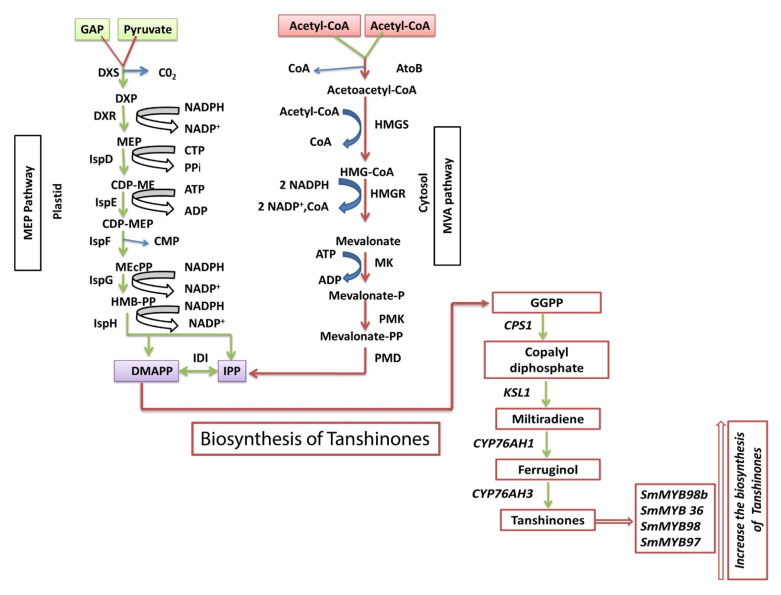
General Biosynthetic pathway of terpenoids (Tanshinones). The red arrow indicates the improvement of biosynthesis of tanshinones through the positive regulation of R2R3 MYB TFs. 2-C-methyl-D-erythritol 4-phosphate (MEP) biosynthetic pathway takes place in the cytosol and mevalonate (MVA) biosynthetic pathway takes place in plastid areas. SmMYB98b, SmMYB36, SmMYB98 and SmMYB97 positively regulate the tanshinones biosynthesis.

Tanshinones are the liposoluble and major bioactive compounds usually present in medicinal herb plants, such as *salvia miltiorrhiza*. Enhancing the production of Tanshinones is critical because of its economic values in human medicine, anti-tumor properties and the curing of cerebrovascular and cardiovascular diseases. Tanshinones has various biological functions, including antiallergic effects, anti-inflammation, anti-cancer, antioxidant and anti-microbial [108,109]. Tanshinones are the diterpenoids, which are synthesized via two different pathways, such as the MEP pathway and MVA pathway, which are localized in plastids and cytosol, respectively (Figure 3). *SmMYB98*, belong to subgroup 22, predominantly expressed in lateral roots of *salvia miltiorrhiza* improved the salvianolic acids and tanshinones in their hairy roots [110]. In Danshen, *SmMYB98b* increased the production of Tanshinones [111]. A significant increase in the production of Tanshinones upon the overexpression of *SmMYB9b* in medicinal plants is observed in Figure 3 [112].

## 10. The Flavonol Biosynthetic Pathway Is Regulated by MYB Transcription Factor

**Figure 4:**
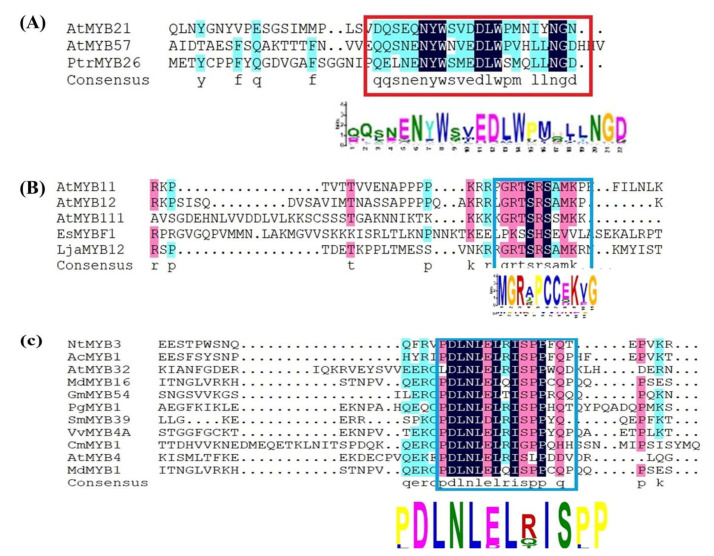
The conserved motif at C-terminal. (**A**) the conserved motif that regulates the anther development; (**B**) this conserved motif regulate the flavonol biosynthesis; (**C**) C2 motif (LxLxLx), which has repressive activities.

The flavonols are colorless and among the most plentiful flavonoids in plants, generally exist in mono-, di-, or triglycerides form [113]. Flavonol regulates several biological functions in plants, such as auxin transport regulation [15,114], the process of fertilization in higher plants, and it is a natural antioxidant [115]. The biosynthetic pathway of flavonol is controlled by MYB protein alone, or acts on the MBW complex or establishment of an MYB-bHLH dimer. A novel gene *PbMYB12b*, positively regulates the flavonol accumulation in pear by up-regulating the *PbFLS* and *PbCHSb* genes [15]. R2R3-MYB transcriptional factor *CcMYB12* isolated from *Cynaracardunculus var. scolymus* and functionally characterized in *Arabidopsis*. Ectopic overexpression of *CcMYB12* activates the levels of gene expression involved in flavonol biosynthesis, ultimately lead to flavonol accumulation in Arabidopsis [116]. In Arabidopsis MYBTFs (*AtMYB111*, *AtMYB11*, and *AtMYB12*) individually accomplished for motivating the genes encoding for flavonol synthase (*FLS*). The expression of *AtMYB12* and *AtMYB111* is spatially differentiated in the emerging seedling. The *AtMYB12* regulates flavonol synthesis generally in the root. Whereas, it was found that *AtMYB111* is dynamic in the cotyledons [113]. Another R2R3-MYB transcriptional factor denoted as *PbMYB12b*, which belongs to subgroup 7, positively regulates the flavonol accumulation in pear fruits [15]. In grapevine, transient assay indicated that *VvMYBF1* controls the expression of *FLS1* and many other promoters of Arabidopsis and grapevine. In *M. truncatula*, R2R3-*MYB134* positive regulator flavanol biosynthesis [117]. Ectopic overexpression of *LjaMYB12* from *Lonicera japonica* enhances the flavonol accumulation in the model plant *Arabidopsis* [7]. Both *GtMYBP3* and *GtMYBP4* genes are isolated from the Japanese gentian (*Gentiana triflora*). When these genes are over-expressed in model plants Arabidopsis, the increase the transcript level of genes encoding for flavonol biosynthetic pathway. Furthermore, flavonol contents were increased in the seedling of Arabidopsis transgenic plants [118]. These previously functionally characterized R2R3-MYB TFs belong to subgroup 7 containing [K/R][R/x][R/K]xGRT[S/x][R/G]xx[M/x]K and ([W/x][L/x]LS) motifs at C-terminal (Figure 4B). *StMTF1* (*Solanum tuberosum*) and *VvMYB5a* (grapevine) belong to subgroup 6 and 27 respectively, involved in the accumulation of flavonol contents [119,120]. *MdMYB3* gene belong to subgroup 4 activate the flavonol biosynthesis [121]. In our previous study, *NtMYB3* and *NtMYB2* are isolated from Chinese narcissus are responsible for the regulation of flavonol biosynthesis. Ectopic overexpression of *NtMYB2* and *NtMYB3* reduced the flavonol contents by suppressing the *FLS* in the transgenic flowers of tobacco [11,12]. In Maize C1 (R2R3 MYB TFs), that functions to accumulate flavonol biosynthesis.

## 11. Expression Pattern of MYB Transcription Factors in Specific Tissues

MYB TFs are constitutively expressed in vegetative and reproductive parts of the plant. Several MYB TFs, which show various expression patterns in specific tissues, are functionally characterized. Some of these MYB TFs, expressed in many tissues and some are involved in the tissue-specific expression. *AtMYB21* and *AtMYB24*, *PsMYB26*, *AmMYB308*, *AmMYB340* are mainly expressed in flowers (Figure 4A). The expression level of *GbMYBF2* was higher in roots as compared to the stem, fruits and leaves [122]. *StMYB12A* mainly expressed in flowers [123]. The highest expression of *GbMYBR1* in Ginkgo was detected in leaves [124]. Maximum expression levels of *NtMYB5* were detected in perianth and corona of Chinese narcissus [125]. The expression level of *SmMYB86* were observed in peel, stem and leaves [126]. The transcript level of *VvMYB4-like* gene was observed in various parts, such as flower, skin, leaves and roots in grapevine [127]. *CsMYB1* from *Crocus sativus* is involved in stigma development and showed expression in stigma tissues [128]. The MYB genes, including *AtMYB26*, *AtMYB57* and *AtMYB103*, are identified [129,130]. In poplar, the existence of *PtrMYB57* was found in all tissues, but not in roots [131]. In *Arabidopsis thaliana*, the down-regulation of *AtMYB103* resulted in aberrant pollens and early tapetal degeneration. In the same way, *AtMYB32* has also been vigorously expressed in papillae, stigma, lateral root primordial and tapetum [130,132]. *NtMYB2* from Chinese narcissus has been involved in the suppression of flavonoid biosynthesis, especially anthocyanin. Our recent study indicated that *NtMYB2* and *NtMYB3* are mainly expressed in the flowers [11,12].

Tissue-specific regulation has also been reported in other plants, *HbMYB1* detected in latex, bark, and leaves of rubber tree while in trapping panel dryness trees, its expression reduced greatly [133]. The expression of *AtMYB101* was limited only to hypocotyls hook and subapical cells of plant. However, in many tissues, *AtMYB65* and *AtMYB33* were co-expressed. The expression pattern of the R2R3-MYB gene is quite unique as indicated by *AtMYB102* in Arabidopsis was down-regulated in stem and up-regulated in young flowers, leaf, and root on treating with ABA [134,135]. *GhMYB9* and *GhMYB7* have been detected in fibers and flowers, and the expression of these gene are developmentally regulated in fibers [136]. Moreover, previous investigations have proposed the involvement of *GaMYB* in seed development, another development, floral initiation and stem elongation [137], it showed a high-level of expression in grass *L. temulentum* in the stamen primordia and floral meristem. In our recent study, we found that *NtMYB4* is involved in development and mainly expressed in flowers (data unpublished). In soybean, *GmMYBJ7* and *GmMYBJ6* were only expressed in stem and leaf, which shows that MYB TF’s common characteristic may represent different pattern among higher plants [138].

## 12. Regulation of Flavonoid Biosynthesis

Recently, we investigated *NtMYB2* and *NtMYB3*, which are isolated from Chinese narcissus and belong to subgroup 4. Ectopic overexpression of these genes in tobacco reduces flavonoid biosynthetic pathway genes that are controlled through the interaction of various families of TFs. The genes which are responsible for anthocyanin biosynthesis are differentially controlled in monocot, as well as dicots species by MBW complex. This MBW complex activates the temporal and spatial transcript of genes (structural), encoding for anthocyanin accumulation. Anthocyanin biosynthesis regulation in monocot differs from dicots species. Several R2R3 MYB (TFs) were recognized in many plants, including *Petunia hybrida*, *Arabidopsis thaliana* and *Zea mays*, which are responsible for flavonoid biosynthesis regulation. Recently plant genome-wide survey provide the opportunities for the identification and isolation of many MYB TFs responsible for regulation of flavonoid biosynthesis form various plant species, including eggplant, strawberry, apple, potato, pear, bayberry, grapevine, pear, poplar, purple kale, soybean, cauliflower and Chinese narcissus [12]. Most of these MYB genes have been functionally characterized by overexpression in host species.

## 13. MYB Transcription Factors Act as Repressors through C2/ERF/TLLFR Motif

**Figure 5:**
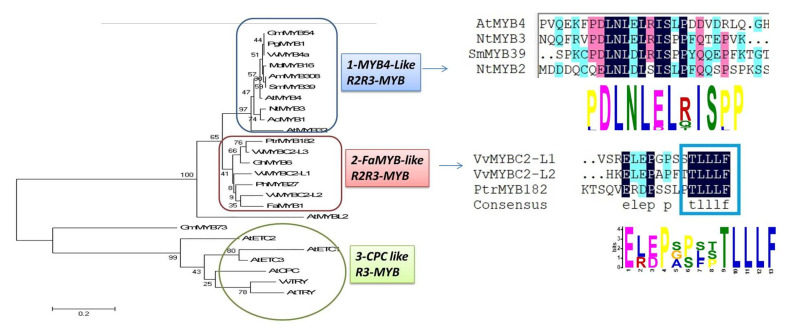
Phylogenetic relationship and signature of conserved protein motifs in R2R3 and R3 MYB repressors connected to phenylpropanoid biosynthesis.

MYB suppressor has conserved the R2R3 region at the end of N-terminal, which also contained the conserved signature of [D/E]Lx2[R/K]x3Lx6Lx3R inside the R3-domain, which interact with *bHLH* cofactor and performed specific functions [139]. For example, *MdMYB15L* could not perform its functional activities without *bHLH* cofactors [140]. MYB TFs show highly divergence at C-terminal domain, which determines the particular functions. Based on diverge protein sequence at C-terminal, they have been divided into subgroups about 22. The proteins of Subgroup 4 function as transcriptional repressors of the phenylpropanoid pathway, and also suppress the key enzymes involved in their metabolic activities. Phylogenetic tree analysis (Figure 5) indicated that R2R3-MYB transcription factor could be divided into three clades, including *FaMYB1-Like*, *MYB4-Like* and *CPC-like* clades (Figure 5). *NtMYB3* and *NtMYB2* anthocyanin repressors are more closely related to the *MYB4-Like clade* [12]. *PtrMYB182* is closely related to the *FaMYB1-like clade*. The MYB repressor belong to *MYB4-Like* clade contain C1 (LlsrGIDPxT /sHRxI /L), also called GIDP motif, which is also found in subgroup 8, 9 and 11 possess activator activities and C2 (LxLxL) or (pdLNLD/ELxiG/S) motif (Figure 2), which is also called EAR motif, and some have C1, C2, C3(CX1-2CX7-12CX2C) and C4 (FLGLx4-7V/LLD/GF/YR/Sx1LMK) motifs depend upon the length of amino acids, but these motifs are absent in *FaMYB1* like Clade, which usually possess TLLLFR motif at their C-terminus (Figure 2). For example, a similar motif was found in *VvMYB4-like* protein [127]. Another difference in the signature of conserved sequence between *AtMYB4-like* clade *FaMYB1-like* clade is the DNEI and DNEV, respectively, which are usually present in R3 domain. The potential and the importance of DENI or DNEV conserved signature in R2R3-MYB repressor proteins are needed for further investigation. The C-terminus of MYB TFs show divergent. They are considered to be accountable for various regulatory functions. Moreover, MYB repressors belong to *AtMYB4-like* clade directly binds on the promoters of targets genes for example *MdMYB16*, while MYB repressor members of *FaMYB-like* clade need MBW complexes for their function, for example *PhMYB27* [141,142]. *SmMYB86* gene was isolate from eggplant and its overexpression reduced the anthocyanin. *SmMYB86* directly binds on promoters and repress the activities of *ANS*, *F3H* and *CHS* [126].

From *Antirrhinum majus*, *AmMYB308* and *AmMYB330*, adversely affected lignin and phenolic acids biosynthesis through its overexpression in tobacco. Furthermore, key enzymes, including *4CL*, *C4H* and *CAD* encoding for the phenylpropanoid pathway, were downregulate [143]. Overexpression of *ZmMYB42* and *ZmMYB31* in *Arabidopsis thaliana* reduced lignin contents [144]. In Arabidopsis, *AtMYB32* and *AtMYB4* knockout mutant enhanced the yield of lignin and sinapate esters by increasing the expression level of the *C4H* gene. In addition, MYB4 acts as a repressor of phenylpropanoid metabolisms by downregulating the expression of genes encoding arogenate Dehydratase 6, which catalyzes the final step in phenylalanine biosynthesis [132,145]. *MdMYB6* isolated from apple, reduced the anthocyanin accumulation upon its overexpression in *Arabidopsis thaliana*. Overexpression of *AtMYB60* in lettuce repress the anthocyanin accumulation by strongly down-regulate the dihydroflavonol 4-reductase (DFR) gene [146]. A dramatic reduction of anthocyanin and flavonols accumulation were observed in transgenic tobacco when *FaMYB1* was overexpressed in tobacco. Proanthocyanidins were also reduced in the leaves of lotus [147]. Albert et al. (2014) described when *FcMYB1* was suppressed in strawberry fruits, the accumulation of anthocyanin to increase in strawberry fruits. When *PhMYB27* repressed by RNA interference increased the anthocyanin accumulation in vegetative tissues and flowers of Petunia. Furthermore, the suppressive action of *PhMYB27* decreased by cut of DLNxxP –type EAR motif. *In grapevine*, *VvMYBC2-L1* is a novel locus, down-regulate the expression of proanthocyanidins pathway-related gene and decreased the amount of PA by overexpressing in hairy root [148]. *AtMYB7*, reduce the biosynthesis of flavonol though directly targeting genes, such as *UGT* and *DFR*, and negatively regulate the seed germination [149,150]. *CmMYB1*, from Chrysanthemum, recognized as repressor candidate, negatively regulate the lignin and flavonoid pathway, reduced the flavonoid and lignin contents in *Arabidopsis thaliana* [151]. Overexpression of *SmMYB39* in Salvia strongly reduced the total phenolics, salvianolic acid A and B, 4-coumaric acid and 4-coumaric acid through downregulation of tyrosine aminotransfersase (TAT) and 4-hydroxylase (C4H) genes [107]. In poplar plants, *PtrMYB57* suppress the anthocyanin and proanthocyanidin accumulation [152]. *TaMYB4* (from wheat) reduced the expression of cinnnamoyl-CoA reductase (CCR) and cinnamyl alcohol dehydrogenase(CAD) in transgenic tobacco [153]. In addition, microRNA 858 and microRNA156 have negative effect on anthocyanin biosynthesis by expressing their target MYB gene [154] (Table 2).

The members of CPC-like clade contain only single R3 repeat, belong to one of 12 different subgroups, which is determined on the base of the conserved motif. The phylogenetic tree divided the R3-MYB repressor into two further subclades, including *CPC-like* and *AtMYBL2-like*, and showed evolutionary derivation from each other (Figure 4). The members of R3-MYB belong to *CPC–like* clade included in a single clade, showed clear divergence from *AtMYBL2 clade* and R2R3-MYB repressors clade. The at *MYBL2-like* clade is closely related to the R2R3-MYB repressor, possesses TLLLFR repressive motif at the C-terminus, but repress transcriptional mechanisms are different from CPC-like clade. WxM motif, involved in cell movement, is present in *CPC-like* clade but absent in *AtMYBL2-like* clade. The *CPC-like* repressors do not contain repressive motif and considered to be act as repressors through competing for *bHLH* cofactor with MYB activators. They interact with *bHLH* cofactors in competitive way to inhibit the creation of MBW complex. Therefore, they negatively control anthocyanin biosynthesis [71]. The evidence from the recent studies showed that R3-MYB suppressors negatively control the anthocyanin biosynthesis like R2R3 MYB suppressors. *IlMYBL1*, is a novel R3-MYB transcriptional repressor, which reduced floral pigmentation in *Iochroma* [171]. In *Arabidopsis*, *ETC1* was concerned with the down regulation of anthocyanin [163,172]. In tomato, *AtMYBL2* encodes an R3-MYB protein that regulates the flavonoid biosynthesis. It comprises of a single repeat and shows resemblance with the R2R3-MYBs, which contrasts with other members of R3-MYBs. In the mutant seedling of *Arabidopsis thaliana*, anthocyanin accumulation was improved due to the loss of *MYBL2* activity. Moreover, overexpression of *AtMYBL2* in seeds led to the suppression of proanthocyanidin accumulation [173]. *AtMYBL2* showed the C-terminal TLLLFR motif in their protein, which contributes to suppressive activities [173,174]. *MYBx* and *PhMYB27*, from Petunia encode a R3-MYB protein suppress the anthocyanin accumulation [163] (Table 2).

## 14. Conclusions and Future Perspective

The MYB transcription factor family is significant in the regulation of bioactive compounds through phenylpropanoid and flavonoid biosynthetic pathway in plants. Generally, it seems that the MYB repressors have more extensive effects than the corresponding MYB activators. Several studies of MYB suppressors exploiting overexpression in heterologous model plant systems, but it is important to authenticate effects using more direct analyses. To date, the role of the conserved motif of repression in MYB TFs is little known. Theses motifs bind and recruits co-repressors or other regulatory proteins for proper functions. Further studies need to emphasis testing the promoters and bHLH binding capacity of more diversity of MYB repressors. Recognizing suppressor binding targets on a whole-genome scale using ChIP-sequence offers further facts on targets. Eventually, a comprehensive understanding of MYB repressors will assist us to better understand the fine transcriptional regulation of the phenylpropanoid pathway and how they facilitate responses to environmental stress. The regulatory system of transcriptional repressors and activators regulating anthocyanin biosynthesis is conserved within monocot species. R3-MYB looks a potentially valuable target for floral color modification in horticulture plants. *S. miltiorrhiza* is a potential medicinal plant, and in order to increase its clinical demand, we need to apply recent advanced metabolic engineering approaches to improve and enhance the biosynthesis of bioactive medicinal compounds.

Several studies on the role of MYB TFs in the regulation of secondary metabolites and bioactive compounds have been done in plants. There is no study in its role in microorganisms (bacteria and yeast) and microalgae. Microalgae has great potential as an expression platform for recombinant proteins. Photosynthetic Microalgae, especially *Chlamydomonas reinhardtii*, is considered as the model host organisms for heterologous protein production, including pharmaceutical products, vaccines, fuels alternative, cosmetics, terpenoids and secondary metabolites. We suggest that there should be more focus on investigating the role of MYB TFs in microalgae for future perspectives, especially regarding the pharmaceutical and food industries. Furthermore, the role of micro-RNA and post translational modification still concern questions to comprehensively understand MYB repressors regulation mechanisms.

## Figures and Tables

**Table 1:** MYB TFs involved in plant defense mechanisms.

Plant	Transcriptional Factors	Plant Defense	References
*Saccharum Spontaneum*	*MYB36*, *MYB48*, *MYB 54*, *MYB61*	Drought stress resistance	[30]
*Arabidopsis thaliana*	*MYB 28*, *MYB29*	ammonium stress	[62]
Plant roots	*MYB41*, *MYB53*, *MYB93*, *MYB92*	Forming protective barrier against biotic and abiotic	[63]
*Saccharum Spontaneum*	MYBs	resistance against mosaic diseases	[30]
*Solanum lycopersicum*	*SlMYB52*	enhancing the tolerance against spider mites by regulating trichome formation	[64]
*Vitis vinifera* L.	*VvMYB1*, *VvMYBA3*	salt stress resistance and drought resistance	[65]
*Oryza sativa*	*OsMYB30*	resistance in rice against brown planthopper	[61]
*Lilium longiflorum*	*LlMYB305*	positive regulator of thermotolerance	[1]

**Table 2:** MYB TFs involved in plant general flavonoid pathway and act as repression functions.

Species	Group	Protein/Gene	Target Gene	Functions	References
*Chrysanthemum*	R2R3-MYB	CmMYB8	*PAL*, *C4H*, *4CL1*, *HCT*, *CCR1*, *AOMT1*, *COMT*	Reducing the contents of Lignin and flavonoids	[155]
*Petunia hybrida*	R2R3-MYB	PhPH4	*F3H*, *F3′H*, *F3*, *F3′*, *5′H*	Anthocyanin Repressor	[69]
*Citrus*	R2R3-MYB	CsMYB3	*CsRuby1/CsbHLH1*	Reduced the anthocyanin	[156]
*Pear*	R2R3-MYB	PbMYB120	*UFGT*	Negative regulator of anthocyanin biosynthesis	[157]
*Arabidopsis*	R2R3-MYB	MYB4	*ADT6*	Phenylpropanoid metabolisms	[145]
*Vitis vinifera*	R2R3-MYB	VvMYB4-like	*ANS*, *DFR*, *UFGT*	Anthocyanin repressor	[127]
*Vitis vinifera*	R2R3-MYB	VvMYBC2L2	*DFR*, *UDP*, *UFGT*, *AN1a*, *AN1b*	Negative regulator of anthocyanin biosynthesis	[158]
*Glycine max*	R2R3-MYB	GmMYB100	*CHS*, *CHI*, *F3H*, *ANS*	Negatively regulate flavonoid biosynthesis	[159]
*Ginkgo biloba*	MYB	GmMYBR1	*GL1*	Anthocyanin, Lignin, Flavonol and Proanthocyanin reduced	[124]
Banana	R2R3-MYB	MaMYB4	*CHS*, *DFR*, *ANS*	Anthocyanin repressor	[160]
*Malus domestica*	R2R3-MYB	MdMYB6, MdMYB16	*ANS*, *UFGT*	Reduce the contents anthocyanin	[141,161]
*Populus tremuloides*	R2R3-MYB	PtrMYB57	*CHS*, *4CL*, *DFR*, *ANS*, *ANR*, *LAR*	Reduction of anthocyanin and Proanthocyanidin	[152]
*Populus tremuloides*	R2R3-MYB	PtrMYB182	*ANR1*, *CHS1*, *DFR*	Down-regulation of anthocyanin and Proanthocyanidin	[132]
*Arabidopsis*	R2R3-MYB	AtMYB3	*C4H*	Phenylpropanoid repressor	[162]
*Arabidopsis*	R2R3-MYB	AtMYB60	*DFR*	Anthocyanin repressor	[146]
*Arabidopsis*	R2R3-MYB	AtMYB7	*UGT*, *DFR*	Suppression of flavonol	[149]
*Arabidopsis*	R3-MYB	CPC	*DFR*, *LDOX*	Down regulation of anthocyanin	[163]
*Arabidopsis*	R3-MYB	AtMYBL2	*DFR*, *LDOX*	Anthocyanin Repressor	[164]
*Fragaria ananassa*	R2R3-MYB	FaMYB1	*DFR*, *ANS*, *ANR*	Reduce the anthocyanin and Flavonol, Proanthocyanidins	[147,165]
*Fragaria chiloensis*	R2R3-MYB	FcMYB1	*LAR*, *ANS*, *ANR*	Anthocyanin repressor	[166]
*Petunia hybrida*	R2R3-MYB	PhMYB27	*ANS*, *F3′5′H*, *DFR*	Anthocyanin repressor	[142]
*Freesia hybrida*	R3-MYB	FhMYBx		Anthocyanin repressor	[167]
*Vitis vinifera*	R2R3-MYB	VvMYBC2-L1		Proanthocyanidins	[168]
*Chrysanthemum morifolium*	R2R3-MYB	CmMYB1	*CHS*, *CHI*, *FLS*, *DFR*	Repressor of lignin and flavonoid	[151]
*Salvia miltiorrhiza*	R2R3-MYB	SmMYB39	*C4H*, *TAT*	Reduce the total phenolics	[107]
*Grape hyacinth*	R3-MYB	MaMYBx	*DFR*, *ANS*	Anthocyanin repressor	[169]
*Narcissus tazetta*	R2R3- MYB	*NtMYB2*	*ANS*, *UFGT*	It reduced the anthocyanin contents and flower pigments	[11]
*Narcissus tazetta*	R2R3- MYB	*NtMYB3*	*DFR*, *UFGT*, *ANS*	It reduced the anthocyanin and flavonol contents. It strongly decreased the flower pigments	[12]
*Populus trichocarpa*	R3-MYB	*PtrRML1*	*DFR*, *UF3GT*	Anthocyanin reduced	[170]
*Chinese Narcissus*	R2R3-MYB	*NtMYB5*	*DFR*, *UFGT*	Suppressor of Anthocyanin	[125]
*Iochroma*	R3-MYB	*MYBL1*	*DFR*, *ANS*, *CHS*, *F3H*	Losses of floral pigmentation	[171]

## Data Availability

Not applicable.
